# Baseline Characteristics and Postdischarge Outcomes by Medication for Opioid Use Disorder Status Among Women with Polysubstance Use in Residential Treatment

**DOI:** 10.1089/whr.2023.0082

**Published:** 2023-12-15

**Authors:** Anna Beth Parlier-Ahmad, Sydney Kelpin, Caitlin E. Martin, Dace S. Svikis

**Affiliations:** ^1^ Department of Psychology, Institute for Women's Health, Virginia Commonwealth University, Richmond, Virginia, USA.; ^2^ Department of Obstetrics and Gynecology, Institute for Drug and Alcohol Studies, Virginia Commonwealth University, Richmond, Virginia, USA.

**Keywords:** medication for opioid use disorder, residential treatment, women, opioid use disorder, buprenorphine, methadone

## Abstract

**Background:**

Within residential treatment, medication for opioid use disorder (MOUD) is rarely offered, so little is known about group differences by MOUD status. This study characterizes samples of women receiving and not receiving MOUD and explores postdischarge outcomes.

**Methods:**

This is a secondary exploratory analysis of a residential clinical trial comparing women receiving treatment as usual (TAU) with those who also received computer-based training for cognitive behavioral therapy (CBT4CBT). Participants were *N* = 41 adult women with substance use disorder (SUD) who self-reported lifetime polysubstance use. Because 59.0% were prescribed MOUD (MOUD *n* = 24, no MOUD *n* = 17), baseline variables were compared by MOUD status; outcomes at 12 weeks postdischarge were compared by MOUD status and treatment condition using chi square and Mann–Whitney *U* tests.

**Results:**

Participants were middle-aged (41.7 ± 11.6 years) and non-Latinx Black (80.4%). Most used substances in the No MOUD group were alcohol, cocaine, and cannabis, and in the MOUD group, most used substances were opioids, cannabis, and cocaine. Women in the MOUD group tended to have more severe SUD. Postdischarge substance use recurrence rates were twice as high in the MOUD group than in the No MOUD group. Among the women in the No MOUD group, those in the CBT4CBT condition increased the number of coping strategies twice as much as those receiving TAU.

**Conclusion:**

Postdischarge substance use recurrence differed by MOUD status. CBT4CBT may be a helpful adjunct to personalized residential SUD treatment. The parent study is registered at [www.clinicaltrials.gov (ClinicalTrials.gov identifier: NCT03678051)].

## Introduction

Historically in the United States, the disease model for substance use disorder (SUD) led to a proliferation of abstinence-focused residential treatment programs with the postdischarge goal of continued abstinence.^1^ In the 1960s, revolutionary physician-scientists introduced the novel concept that addiction is a chronic disease requiring long-term treatment.^2,3^ Despite research showing medication to be an effective long-term treatment for SUD, it has been stigmatized by the misconception that it “substitutes one addiction for another.” Therefore, traditionally patients with SUD, such as opioid use disorder (OUD), were left no other option than medical detoxification before or during admission to residential care.^2,4^

Today, medication for opioid use disorder (MOUD) is the gold standard treatment for OUD. Methadone and buprenorphine are two of the most common types of MOUD. These medications work as opioid agonists, and when taken as prescribed, they provide stability from the physiological effects of OUD, creating a foundation that empowers people with OUD to achieve recovery. Specifically, MOUD reduces euphoric effects of opioids, alleviates withdrawal symptoms, lessens physiological cravings, and normalizes brain chemistry.^5^

Rigorous research demonstrates that MOUD reduces overdose risk on a population level by >50% and is the most effective treatment for OUD.^5,6^ With this updated understanding of how to effectively treat OUD in combination with the rise of the opioid overdose epidemic, there was a paradigm shift within the residential care setting. FDA approval of buprenorphine in addition to methadone for OUD led to increased ease of providing MOUD in this setting and greater acceptance of MOUD as another component of residential SUD treatment. However, the uptake of MOUD remains slow. Currently, less than one in five patients with OUD receive MOUD during residential SUD treatment.^7,8^

Although prior research supports the efficacy of personalized multimodal SUD treatments combining both medication and behavioral treatments, this research has been conducted primarily in outpatient SUD treatment settings.^9^ To date, little is known about postdischarge outcomes for individuals receiving MOUD as a component of their SUD residential treatment experience. Some empirical data have come from perinatal addiction treatment programs, where in the interest of maternal and fetal safety, MOUD has been offered as an adjunct to residential and intensive outpatient SUD treatment.^10^

In a series of studies, investigators found better residential treatment retention for pregnant women prescribed MOUD^11^ and better postdischarge outpatient treatment participation and retention in participants receiving MOUD than those not receiving MOUD.^12^ Unfortunately, outside of pregnancy, SUD treatment is still mostly siloed by MOUD status contributing to a gap in knowledge regarding differences in treatment outcomes.

Identifying differences between women receiving and not receiving MOUD in residential treatment is an important step toward personalizing treatment plans and improving outcomes. Women with SUD are a subpopulation of particular interest as they are less likely to seek SUD treatment than men and face unique barriers to care including childcare responsibilities, fear of legal consequences, lack of transportation, financial limitations, and stigma.^13–15^

In a nationally representative sample, women were more likely than men to report worry about their reputation or job as well as transportation and/or time barriers to SUD treatment.^16^ Women also experience higher rates of co-occurring mental health conditions than men such as mood, eating, anxiety, and trauma-related disorders. Such comorbidities also create barriers to receiving appropriate services.^14,17^

When women do present to treatment, they tend to have more medical, psychiatric, and social vulnerabilities than their male counterparts.^18^ Prior research has found that gender-specific treatment programs have higher retention rates, less substance use, and fewer barriers to care.^19^ However, even in such programs, substance use recurrence rates remain high, with 35%–60% of people returning to nonprescribed substance use.^20,21^

Depression, interpersonal stress, and relationship conflict are more likely to be associated with substance use recurrence in women than in men.^13,22^ Therefore, in addition to gender-specific treatment, tailored gender-informed care is essential to improving SUD outcomes. For example, teaching coping skills and stress management techniques as part of gender-informed SUD treatment may be central to preventing substance use recurrence specifically among women.

The parent clinical trial for this study examined feasibility of providing computerized cognitive behavioral therapy to women with SUD in residential treatment, a treatment setting wherein polysubstance use and severe SUD are common.^23^ At the time of the randomized controlled trial (RCT), MOUD had been integrated into treatment and was offered to patients with OUD at the residential center where the study took place. Surprisingly, over half of the sample was receiving MOUD, which is strikingly different from prior research.^7,8^

Previous studies show that patients receiving MOUD typically differ from those not receiving MOUD in demographic characteristics and health-related factors as well as SUD history and treatment outcomes; those receiving MOUD are more likely to be non-Latinx White, younger, have fewer comorbidities, and more previous SUD treatment episodes.^11,24,25^ Therefore, stratifying samples by MOUD status is warranted.

Because 59% of RCT participants were receiving MOUD at discharge from residential treatment, this study aimed to (1) describe demographic, psychosocial, and SUD factors of women with a history of polysubstance use receiving and not receiving MOUD and (2) explore postdischarge outcomes within the MOUD and No MOUD groups between those assigned to the intervention study condition and those assigned to treatment as usual (TAU).

To our knowledge, this study is one of the first to characterize samples of women receiving and not receiving MOUD in residential SUD treatment and provide postdischarge data, as well as the first to assess computer-based training for cognitive behavioral therapy (CBT4CBT) within MOUD and No MOUD groups in residential SUD treatment.

## Methods

### Participants

Participants were women admitted to a residential SUD treatment program who were English speaking, ≥18 years of age, met *DSM-5* criteria for SUD (current), and expected to have a residential length of stay ≥4 weeks. Exclusion criteria included currently pregnant, significant cognitive or psychiatric impairment, or language barrier preventing informed consent.

Recruitment occurred from October 2018 to April 2019. Three-fourths (77%) of women approached met study criteria, provided informed consent, and completed the baseline assessment during their first week of treatment.^23^ Participants were then randomized to either the intervention (CBT4CBT; *n* = 34) or control (TAU; *n* = 29) groups. The university's institutional review board approved all study procedures (HM20012674).

Because over half of the sample was receiving MOUD at discharge, this secondary analysis explored postdischarge outcomes separately for women receiving and not receiving MOUD. Data analyses focused on “study completers,” defined as women who completed study staff (research coordinator and assistants) administered assessments through at least 4 weeks postdischarge. All study participants endorsed lifetime polysubstance use, operationalized as self-reported nonprescribed use of at least two substances (lifetime) including opioids, stimulants, sedatives, cannabis, hallucinogens, inhalants, and alcohol. Overall, *N* = 41 participants (MOUD *n* = 24, No MOUD *n* = 17) were included. Within the MOUD group, *n* = 23 were receiving MOUD at baseline and *n* = 1 initiated MOUD during residential SUD treatment.

### Residential treatment program

The study site was a community-based residential SUD treatment program located in a mid-Atlantic U.S. city. All services offered through the residential SUD treatment program were available to study participants regardless of RCT group assignment. Services included individual and group counseling, educational skill building, occupational or recreational activities, and personalized case management (*e.g.*, housing, transportation, and childcare). Counseling services covered a range of topics, including relapse prevention, re-entry skills, health and wellness, relationships, anger and conflict management, leadership skills, intimate partner violence, sexual abuse, and parenting. The program tailored the intensity of treatment services to the patient's needs and used random drug testing to monitor treatment gains.

Buprenorphine and methadone were available as MOUD options during residential treatment. Both were provided by a nurse via daily dose dispensing at the residential treatment center. For buprenorphine, an on-site addiction medicine provider prescribed the medication. Individuals who were prescribed buprenorphine by an outside provider at admission were able to continue their prescription. Individuals not receiving MOUD and experiencing opioid withdrawal were offered buprenorphine induction upon admission to treatment.

For methadone, the residential treatment clinic coordinated with local methadone clinics to have methadone prescribed and transported to the residential treatment facility weekly. Individuals had to be prescribed methadone before admission to receive methadone during residential treatment due to prescribing restrictions.

### Study conditions

#### CBT4CBT condition

Participants randomized to the CBT4CBT condition had access to the individual computer-administered CBT4CBT program in a private area on-site. Study staff scheduled a minimum of two CBT4CBT sessions per week postrandomization and were available during scheduled sessions to help participants with program login and any technological issues. The CBT4CBT program consists of seven modules covering core CBT topics, ∼45 minutes each.^26^ Each module includes on-screen narration, video demonstration of skills, interactive exercises, and practice exercises for between modules (*e.g.*, “homework”).

#### Treatment as usual

In the TAU condition, participants were offered standard residential treatment for SUD at the study clinic. Participants in TAU condition completed the same assessments as those in the CBT4CBT condition.

### Baseline assessments

Baseline measures included demographics (age, race, ethnicity, employment, education, marital status, and living arrangement) and self-reported substance use problems. *DSM-5* diagnoses of alcohol and other drug use disorders were made using the Mini-International Neuropsychiatric Interview.^27^ The alcohol and drug use sections of the MINI required 20–30 minutes to administer. The Addiction Severity Index (ASI) evaluated domains commonly affected by substance use including medical, employment/self-support, alcohol use, drug use, legal status, family–social environment, and psychiatric status.^28^

The ASI required ∼45 minutes to administer. The lifetime substance use variable is based on self-reported ASI data. Craving for problematic nonprescribed substance was assessed using the 3-item Brief Substance Craving Scale (BCBS) that measures the intensity, frequency, and length of cravings during the past 24 hours using a 5-point Likert scale (0–4).^29^ Items are summed to yield an overall measure of craving ranging from 0 to 12 with higher scores indicating worse cravings.

The 20-item Center for Epidemiological Studies Depression Scale (CES-D) assessed depressive symptoms over the past week using a 5-point Likert scale (0 = rarely or none of the time and 4 = most or all of the time).^30^ Scores range from 0 to 60; a score ≥16 indicates probable depression. The 7-item Generalized Anxiety Disorder (GAD-7) scale assessed anxiety symptoms over the past 2 weeks using a 4-point Likert scale (0 = not at all and 3 = nearly every day).^31^ Scores range from 0 to 21; a score of ≥10 represents a probable Generalized Anxiety Disorder diagnosis.

The 10-item Perceived Stress Scale (PSS) measured stress over the past month using a 5-point Likert scale (0 = never, 4 = almost always).^32^ Summed scores range from 0 to 40 and are categorized as low (range 0–13), moderate (range 14–26), or high (range 27–40) stress.

### Postdischarge outcomes

For the current analyses, postdischarge outcomes were assessed either in-person or virtually over the 12-week postdischarge period (measured at both 4- and 12-week follow-up visits).

The primary outcome was presence of any nonprescribed substance use recurrence during the 12-week postdischarge period. Any substance use recurrence was defined as self-reported nonprescribed substance use (alcohol or other drugs) on the Timeline Follow Back (TLFB), a calendar method used to collect daily alcohol and nonprescribed drug use data.^33^ Participants missing data after the 4-week follow-up (*n* = 6) were considered relapsed on the day after the last point of contact with the participant.

The secondary outcome was change in number of coping strategies utilized between treatment entry and 12 weeks postdischarge. Frequency of employing coping strategies over the past week (5-point Likert scale; 0 = never, 4 = all the time) was assessed using the Sugarman et al. 17-item Coping Strategies Scale (CSS-17) including both alcohol and other drugs (Cronbach's *α* = 0.82); scores range from 0 to 68 with higher scores indicating more coping strategies utilized. Change in number of coping strategies was operationalized as the difference in the number of strategies reported at baseline and 12-week follow-up visit. For participants missing 12-week follow-up data, 4-week CSS data were used (*n* = 6).

### Data analyses

Baseline demographic, psychosocial, and SUD factors were compared by MOUD status using chi square or Fisher exact tests for categorical variables and *t*-test or Mann–Whitney *U* for continuous variables. Substance use recurrence for any substance (yes/no) was compared between MOUD and No MOUD groups using chi square analysis. In addition, substance use recurrence for any substance (yes/no) and change in average number of coping strategies utilized (range 0–68) were compared between the two study arms (CBT4CBT vs. TAU) within the MOUD groups using chi square and *t*-tests, respectively. To account for multiple comparisons, Bonferroni correction was used, and significance was set at 0.002.

## Results

Participants (*N* = 41) were primarily middle aged (41.7 ± 11.6 years), non-Latinx Black (80.4%), and single (73.2%). Approximately half of participants were unemployed or receiving disability. Most participants had a history of psychiatric problems (92.7%). Based on current symptoms, over half (58.5%) endorsed probable anxiety, and three in four endorsed probable depression (75.6%). Most participants reported moderate-to-high stress (87.8%) and utilized few coping strategies at baseline [median = 8 (interquartile range 0–20)]. Participants did not differ in demographic or psychosocial variables by MOUD status (Table 1).

**Table 1.** Demographic, Psychosocial, and Substance Use Disorder Factors of Study Completers by Medication for Opioid Use Disorder Status

Baseline demographic, psychosocial, and SUD factors	MOUD,^a^ ***n*** (%), ***N*** = 24	No MOUD,^a^ ***n*** (%), ***N*** = 17	***p***
Age (mean ± SD)	39.3 ± 11.0	45.1 ± 12.0	0.116
Race			0.575
Black	19 (79.2)	15 (88.2)	
White	4 (16.7)	1 (5.9)	
Other	1 (4.2)	1 (5.9)	
Ethnicity			0.663
Non-Latinx	23 (95.8)	16 (94.1)	
Latinx	1 (4.2)	1 (5.9)	
Employment			0.384
Unemployed/disability	12 (50.0)	10 (58.8)	
Employed	9 (37.5)	6 (35.3)	
Homemaker	3 (12.5)	1 (5.9)	
Education			0.342
<High school degree	4 (16.7)	4 (23.5)	
High school degree/equivalent	14 (58.3)	6 (35.3)	
>High school degree	6 (25.0)	7 (41.2)	
Marital status			0.793
Single	18 (75.0)	12 (70.6)	
Married/in a relationship	3 (12.5)	2 (11.8)	
Divorced/separated	2 (8.3)	2 (11.8)	
Widowed	1 (4.2)	1 (5.9)	
Living arrangement at treatment entry			0.075
With a sexual partner	4 (16.7)	0	
With a sexual partner and children	1 (4.2)	2 (11.8)	
With children alone	3 (12.5)	0	
Alone	3 (12.5)	5 (29.4)	
With family/friends	6 (25.0)	8 (47.1)	
Unhoused	7 (29.2)	2 (11.8)	
Ever experienced severe psychiatric problems	22 (91.7)	16 (94.1)	0.630
Ever prescribed psychiatric medication	19 (79.2)	14 (82.4)	0.563
Ever experienced suicidal ideation or had a suicide attempt	11 (45.8)	11 (64.7)	0.233
CESD probable depression (≥16)	20 (83.3)	11 (64.7)	0.159
GAD probable anxiety (≥10)	13 (54.2)	11 (64.7)	0.500
Perceived stress			0.207
Low	4 (16.7)	1 (5.9)	
Moderate	9 (37.5)	11 (64.7)	
High	11 (45.8)	5 (29.4)	
CSS total score^b^ (mean ± SD)	13.2 ± 15.7	13.7 ± 16.7	0.748
Lifetime nonprescribed substance use			
Opioids	24 (100)	5 (29.4)	<**0.001**
Cocaine	20 (83.3)	15 (88.2)	0.512
Alcohol	14 (58.3)	16 (94.1)	0.011
Cannabis	22 (91.7)	13 (76.5)	0.182
Other (sedatives, amphetamines, hallucinogens/inhalants)	12 (50.0)	1 (5.9)	0.003
Self-reported problems with nonprescribed substance			0.015
Opioids only	3 (12.5)	0	
Cocaine only	1 (4.2)	3 (17.6)	
Alcohol only	0	4 (23.5)	
Multiple substances	20 (83.3)	10 (58.8)	
Prior SUD treatment	20 (83.3)	14 (82.4)	0.626
SUD treatment prompted by legal system	7 (29.2)	3 (17.6)	0.321
Injection drug use	13 (54.2)	0	**<0.001**
Prior overdose	10 (41.7)	4 (23.5)	0.192
Currently living with someone with drug and/or alcohol problems	4 (16.7)	3 (17.6)	0.626
BSCS total score^c^ (mean ± SD)	11.2 ± 1.3	10.3 ± 2.2	0.169

Bold indicates significance at *p* = .002.

^a^ Due to rounding to the tenths place value, some total percentages add to 100.1%.

^b^ CSS scores range from 0 to 68 with higher scores indicating more coping strategies.

^c^ BSCS scores range from 0 to 12 with higher scores indicating worse craving.

BSCS, Brief Substance Craving Scale; CESD, Center for Epidemiological Studies Depression Scale; CSS, Coping Strategies Scale; GAD, Generalized Anxiety Disorder; MOUD, medication for opioid use disorder; SD, standard deviation; SUD, substance use disorder.

Nearly all women with OUD were receiving MOUD (92.3%). Of those receiving MOUD, 91.7% (*n* = 22) received buprenorphine and 8.3% (*n* = 2) received methadone. In the MOUD group, the most common substances used were opioids, cannabis, and cocaine (Table 1). In the No MOUD group, the most common substances used were alcohol, cocaine, and cannabis. Participants in the MOUD group had higher rates of injection drug use (*p* < 0.001) than the No MOUD group, and a greater percentage in the MOUD group reported prior overdose and problems with more than one substance (Table 1).

Of those randomized to CBT4CBT, 81.8% (*n* = 9) of the participants in the No MOUD group and 72.7% (*n* = 8) of participants in the MOUD group completed six of seven modules. On average, the No MOUD group completed approximately one more CBT4CBT module than the MOUD group (No MOUD: 6.1 ± 1.8 vs. MOUD: 4.9 ± 3.2).

Overall, the MOUD group had rates of any nonprescribed substance use recurrence nearly twice as high as the No MOUD group [58.8% (*n* = 14) vs. 29.4% (*n* = 5), *χ*^2^(1) = 3.35, *p* = 0.067]; this difference was not statistically significant. Among those with a recurrence in the MOUD group, eight (57.1%) had an opioid use recurrence, eight (57.1%) had cocaine use recurrence, five (35.7%) had cannabis use recurrence, four (28.6%) had alcohol use recurrence, and seven (50.0%) had polysubstance use recurrence.

**FIG. 1.**
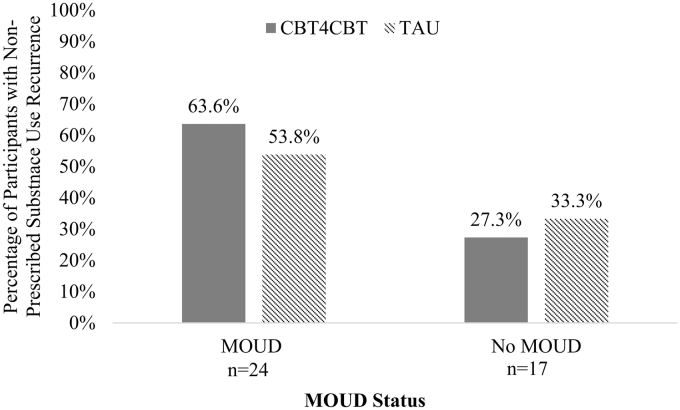
Percentage of participants with any nonprescribed substance use recurrence within MOUD status by treatment condition (*n* = 41). MOUD, medication for opioid use disorder.

Of the participants with opioid use recurrence, *n* = 5 (62.5%) had a lapse in MOUD during the 12-week postdischarge period. Among those with a recurrence in the No MOUD group, three (60.0%) had cocaine use recurrence, three (60.0%) had alcohol use recurrence, one (20.0%) had cannabis use recurrence, and two (40.0%) had polysubstance use recurrence. Nonprescribed substance use recurrence did not differ significantly by treatment condition within the MOUD or No MOUD groups for any substance use (MOUD: 63.6% CBT4CBT vs. 53.8% TAU, *χ*^2^(1) = 0.24, *p* = 0.473; No MOUD: 27.3% CBT4CBT vs. 33.3% TAU, *χ*^2^(1) = 0.07, *p* = 0.605; Fig. 1).

**FIG. 2.**
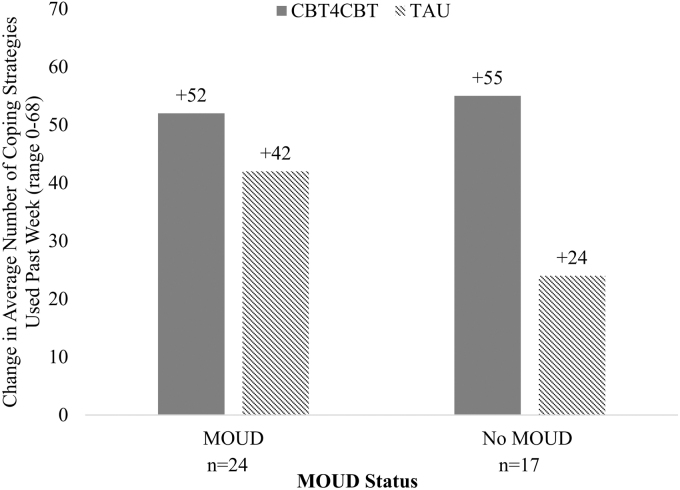
Change in the average number of coping strategies between 12 weeks postdischarge and baseline within MOUD status by treatment condition (*n* = 41).

Within both the MOUD and No MOUD groups, participants in the CBT4CBT condition showed a greater increase in the number of coping strategies used than those in the TAU condition, but this difference did not reach significance after correction [MOUD: 51.5 ± 13.4 vs. 41.6 ± 21.7, *t*(22) = 1.3, *p* = 0.202; No MOUD: 55.2 ± 16.6 vs. 23.5 ± 22.9, *t*(15) = 3.3, *p* = 0.005; Fig. 2].

## Discussion

This study utilized data from a pilot clinical trial conducted in a women's residential SUD treatment program where MOUD was available for individuals with OUD. Therefore, the parent clinical trial provided a unique opportunity to characterize women receiving and not receiving MOUD enrolled in the same treatment program. In addition, we examined postdischarge outcomes within the MOUD and No MOUD groups. To our knowledge, this study is the first to make such comparisons and provide benchmark data to inform future research.

In this study, demographic and psychosocial factors were similar between the MOUD and No MOUD groups. However, our findings suggest that further research is needed to investigate potential differences in SUD variables. Contrasting prior research, we found that nearly all women with OUD were receiving MOUD.^7,8^ In the No MOUD group, approximately one in three participants reported use of opioids during their lifetime compared with all participants in the MOUD group.

Most participants in the No MOUD group reported lifetime use of alcohol and cocaine. Findings suggest that the MOUD group likely experienced more severe SUD as evidenced by higher proportions of injection drug use, multiple problematic substances, and prior overdose than the No MOUD group. With the increase of MOUD in residential SUD treatment settings, those receiving and not receiving MOUD may look increasingly different, underscoring the need to examine these groups separately when designing research studies and tailoring SUD treatment plans.

Because few residential treatment centers offer MOUD and research attrition rates are high in this setting, documented recurrence rates postdischarge from residential treatment among those receiving MOUD are limited.^34^ Prior research on substance use recurrence within outpatient and short-term inpatient or detoxification settings has identified a wide range of recurrence rate estimates (∼35%–60%).^20,21^ In this study, women in the MOUD group had rates of recurrences at the higher end of the range seen in the literature (59%), whereas the No MOUD group reported rates of recurrence below previous estimates (29%).

The difference in substance use recurrence by MOUD status is likely due to several psychosocial and substance use factors. First, over half of the women in the MOUD group with a recurrence had an opioid use recurrence. Many of these women specifically had a recurrence of nonprescribed opioid use coinciding with a MOUD lapse or discontinuation. MOUD is consistently associated with reduced substance use.^6^ However, 35%–45% of people who initiate MOUD discontinue treatment within 6 months.^35^

After discharge from residential treatment, women receiving MOUD may be at especially high risk of discontinuation given that many face psychosocial barriers to access and utilization of MOUD during the postdischarge transition.^13^ Specifically, women often have to overcome logistical barriers such as transportation challenges and lack of insurance, parenting issues including the stress of childcare responsibilities and stigma from support systems and treatment providers, as well as barriers associated with comorbid psychiatric and trauma-related conditions to maintain MOUD postdischarge.^14^

In addition, racial disparities impact MOUD continuation. Black women are at greater risk of MOUD discontinuation than White women,^25^ largely due to long-standing racial inequities in addiction treatment and structural racism.^36^ Residential treatment facilities could help address some of these barriers (*e.g.*, appointment scheduling difficulties, lack of insurance, and transportation challenges) proactively to facilitate continuity of MOUD during the transition from the controlled environment into the “real-world” setting when patients are at high risk of substance use recurrence and overdose.^34^

In addition, future studies of CBT4CBT among MOUD samples in residential treatment should incorporate medication adherence strategies to help equip patients to overcome access barriers and mitigate harm during MOUD lapses. One previous study has demonstrated success in an outpatient SUD treatment clinic with the CBT4CBT–buprenophine program that incorporates an additional module covering the basics of buprenorphine treatment, including strategies for improving buprenorphine maintenance.^37^ Residential treatment centers need to prioritize facilitation of MOUD continuity postdischarge, a particularly vulnerable period.

Although MOUD effectively treats OUD, treatment options for other SUDs, such as stimulant use disorders, remain limited. Recent population-based data demonstrate widespread polysubstance use with a significant rise in overdose deaths involving opioids and stimulants.^38^ In our sample, a greater percentage of women in our MOUD group reported problems with more than one substance than those in our No MOUD group. Over half of the women in the MOUD group with a recurrence had a cocaine use recurrence, and polysubstance use recurrence was common.

Similarly, a secondary analysis of clinical trial data across 13 opioid treatment programs found nearly half of participants reported stimulant use after initiating MOUD, and stimulant use was associated with increased risk of opioid use recurrence.^39^ Our findings alongside recent trends in the literature underscore the importance of providing tailored multimodal treatment to address multiple SUDs and reduce polysubstance use recurrence.

To date, CBT4CBT studies have been conducted in primarily outpatient samples with a subset targeting participants receiving MOUD.^37,40^ In these studies, rates of substance use recurrence were lower in CBT4CBT groups than in those in the TAU condition. In our sample, there was no significant difference in nonprescribed substance use recurrence by treatment condition within the MOUD or No MOUD groups. Although our null findings differed from previous studies, the small parent trial was not powered to detect an effect between treatment conditions stratified by MOUD status.

Future adequately powered studies assessing the impact of CBT4CBT on postdischarge outcomes stratified by MOUD status are warranted given the high-risk nature of the postdischarge period unique to women receiving MOUD in residential treatment settings.

Even with the small sample size, women receiving CBT4CBT in both MOUD groups reported a greater increase in coping strategies than women receiving TAU (though not significant). This pattern of results is consistent with previous CBT4CBT research^41^ and is notable because the coping skills taught in CBT4CBT are directly applicable to common recurrence triggers among women.^13,22^ Given that SUD is a chronic recurring condition, adjunctive CBT4CBT may be a helpful gender-informed strategy in tailored residential treatment plans to teach valuable coping skills, particularly among women not receiving MOUD who may require the use of more behavioral coping strategies to help manage their cravings and reduce or prevent substance use than those receiving MOUD.

### Limitations

Because this was a secondary exploratory analysis of a pilot trial, the study was not powered to detect an effect between treatment conditions stratified by MOUD status; as a result, findings are descriptive. However, consistent with the parent study, no significant difference was identified in postdischarge substance use recurrence between treatment conditions within either MOUD group.^23^ Our relatively high rate of participants receiving MOUD may be a result of selection bias; MOUD receipt is typically associated with better treatment outcomes, so those receiving MOUD may be more stabilized earlier in treatment and more willing to participate in research.

Future studies with larger samples can build upon this study by exploring differences in outcomes by type of MOUD. Notably, our sample is from one residential treatment program in a mid-Atlantic U.S. city; therefore, findings may not generalize to other geographical locations. Although both biological and self-report measures of substance use were in the protocol, many women were unable to complete face-to-face visits due to moving out of the area or having limited transportation, which necessitated use of virtual self-report follow-up data.

Validity of self-reported substance use is variable depending on substance, reporting timeframe, and population; however, prior studies have found strong associations between self-reported and biological measures of substance use.^42^ Future studies would benefit from combining multiple sources of information to improve substance use detection. In addition, we used single imputation (*i.e.*, recurrence) for participants who were missing 12-week follow-up data.^43^ Therefore, we may have overestimated recurrence rates in our sample.

Despite these limitations, our study has several strengths including the makeup of our sample and the longitudinal nature of our postdischarge outcome data. Because we included women with a history of polysubstance use, an under-researched group, our sample is more representative of women in residential SUD treatment than samples limiting participants to one specific type of SUD. In addition, studies assessing residential SUD treatment postdischarge outcomes typically have high attrition rates limiting generalizability. Importantly, we were able to follow 85% of our participants for 12 weeks postdischarge, which reduced the likelihood of attrition bias in our findings.

## Conclusions

Stratifying analyses by MOUD status can help inform future research and clinical practice for women in residential SUD treatment. Postdischarge substance use recurrence differed by MOUD status, highlighting the unique barriers to continuity of MOUD and the high-risk nature of the postdischarge period among women receiving MOUD. Among women with polysubstance use in residential SUD treatment, CBT4CBT may be a helpful adjunct to personalized treatment plans, particularly for those not receiving MOUD.

## Abbreviations Used

BCBS: Brief Substance Craving Scale
CBT4CBT: computer-based training for cognitive behavioral therapy
CSS: Coping Strategies Scale
GAD: Generalized Anxiety Disorder
MOUD: medication for opioid use disorder
RCT: randomized controlled trial
SD: standard deviation
SUD: substance use disorder
TAU: treatment as usual
